# Factors associated with survival after pediatric in-hospital cardiac arrest: A national database analysis 2015–2022

**DOI:** 10.1371/journal.pone.0341430

**Published:** 2026-03-02

**Authors:** Rattapon Uppala, Phanthila Sitthikarnkha, Sirapoom Niamsanit, Kaewjai Thepsuthammarat, Leelawadee Techasatian, Suchaorn Saengnipanthkul, Pope Kosararaksa, Sysavanh Nanthavongsa, Akihiro Nishi

**Affiliations:** 1 Department of Pediatrics, Faculty of Medicine, Khon Kaen University, Khon Kaen, Thailand; 2 Clinical Epidemiology Unit, Faculty of Medicine, Khon Kaen University, Khon Kaen, Thailand; 3 Department of Epidemiology, University of California, Los Angeles Fielding School of Public Health, Los Angeles, California, United States of America; 4 California Center for Population Research, UCLA, Los Angeles, California, United States of America; Azienda Ospedaliero Universitaria Careggi, ITALY

## Abstract

**Background:**

Pediatric in-hospital cardiac arrest (IHCA) is frequently fatal, and evidence from middle-income settings needed to guide quality improvement is sparse. We used nationwide Thai data to quantify incidence and mortality trends, describe long-term outcomes, and identify associated factors for post-discharge death after pediatric IHCA.

**Methods:**

We performed a retrospective cohort study using the Thai National Health Security Office database encompassing all hospitalizations under the universal health-coverage scheme from 1 January 2015–31 December 2022. Children <18 years with IHCA were identified by ICD-10-TM codes I46.0/I46.1/I46.9 plus at least one resuscitation procedure code (ICD-9-CM 99.60/99.62/99.63). All-cause mortality through 31 December 2023 was obtained via linkage to the national civil registry.

**Results:**

Among 13.2 million pediatric admissions, 20,590 IHCAs were recorded (incidence 1.57/1,000). Incidence declined from 1.8/1,000 in 2015–2016 to 1.2/1,000 in 2022. In-hospital mortality was 62.7% (12,905/20,590). Of 7,253 survivors with follow-up (median 67 months), 2,149 (29.6%) died post-discharge. Multivariable analysis identified metabolic acidosis (adjusted hazard ratio [aHR] 1.50; 95% confidence interval [CI] 1.32–1.71) and hypoglycemia (aHR 1.54; 95% CI 1.25–1.89) as significant associated diagnoses with long-term mortality. Furthermore, diagnoses consistent with severe organ dysfunction, including disseminated intravascular coagulation (aHR 1.51; 95% CI 1.24–1.85), acute liver failure (aHR 1.42; 95% CI 1.10–1.84), and anoxic brain injury (aHR 1.23; 95% CI 1.05–1.44), were also significantly correlated with increased mortality; however, the timing of these diagnoses relative to the cardiac arrest could not be determined.

**Conclusions:**

Pediatric IHCA in Thailand remains highly fatal despite recent declines in incidence and in-hospital mortality. During a median follow-up of 67 months, nearly one-third of survivors died after discharge, underscoring the substantial long-term mortality burden. Metabolic derangements and organ dysfunction were strongly associated with post-discharge mortality, highlighting the need for targeted strategies to improve both survival and long-term outcomes.

## Introduction

Cardiac arrest in pediatric populations remains a critical global health concern due to its high mortality and profound morbidity among survivors [1]. Children present unique physiological challenges during both the pre‐arrest and resuscitation periods, resulting in varied etiologies and outcomes for in‐hospital cardiac arrest (IHCA) [2–4]. Recent data suggest that while the incidence of IHCA in children may be lower than in adults, the mortality rate can be exceedingly high, especially in resource‐limited settings [5].

Advances in pediatric critical care, including more sophisticated monitoring and rapid‐response systems, have contributed to improved survival in certain centers [6–8]. However, this benefit is not uniformly shared across all healthcare settings. In resource‐rich hospitals, dedicated pediatric rapid‐response teams (RRTs) and standardized resuscitation protocols have significantly reduced in‐hospital mortality, whereas facilities with limited pediatric expertise continue to face higher mortality rates [9].

In low- and middle-income countries, limited critical care resources, inconsistent practices, and delayed detection and resuscitation further complicate pediatric IHCA care [10–12]. These factors contribute to poor outcomes for critically ill children, with delays in diagnosis and high mortality rates [13–15]. While single‐center reports can shed light on specific case mixes or best‐practice environments, nationwide data better capture the heterogeneity in healthcare quality, resource allocation, and patient complexity. Findings from such an analysis can help policymakers and clinicians identify the most vulnerable pediatric groups and the critical points during hospitalization that require improvements in recognition, prevention, and resuscitation.

Accordingly, this study leverages nationwide data to quantify incidence, mortality trends, and long-term outcomes. Moreover, we identified associated factors for post-discharge death after pediatric IHCA. Understanding these patterns is essential for designing targeted interventions, optimizing resource utilization, and ultimately improving pediatric IHCA outcomes in Thailand and comparable healthcare systems around the world.

## Materials and methods

### Study design and data source

This study utilized retrospective national data obtained from the Thai National Health Security Office (NHSO) database, which contains anonymized patient information. The NHSO is responsible for overseeing and reimbursing healthcare facilities providing services to Thai citizens under the universal health coverage scheme. The NHSO database encompassed several key data points: 1) the principal diagnosis, indicating the primary reason for hospitalization; 2) co-morbidities, representing underlying patient conditions prior to admission; 3) complications, which are conditions resulting after admission; (4) treatments, including procedures and interventions administered during hospitalization; (5) patient status at discharge as alive or dead; and (6) the cause of death. Although the NHSO database distinguishes principal diagnoses, comorbidities, and complications, it does not contain timestamps for individual diagnoses or complications. Therefore, the temporal sequence between cardiac arrest and recorded diagnoses cannot be determined, and these conditions should not be interpreted as definitively occurring before or after the arrest event. This study was approved by the Khon Kaen University Human Research Ethics Committee under reference number HE681045, with a waiver of informed consent due to the use of de-identified data. The dataset was collected between February 15th and April 30th, 2025. This study followed the guidelines outlined by the Strengthening the Reporting of Observational Studies in Epidemiology (STROBE) statement [16].

### Study population

This study focused on data of children under 18 years of age who were hospitalized and experienced in-hospital cardiac arrest from January 2015 to December 2022. Cardiac arrest in children is defined as the cessation of cardiac mechanical activity, indicated by the absence of a palpable central pulse, unresponsiveness, and apnea, which aligns with the Clinical Practice Guidelines for Pediatric Advanced Life Support [17]. Instances of in-hospital arrest were identified from the complications section of patient records, which specifies events occurring after admission. The diagnoses used for this study were based on codes from the International Statistical Classification of Diseases and Related Health Problems, 10th Revision, Thai Modification (ICD-10-TM) as follows: I46.0 (cardiac arrest with successful resuscitation), I46.1 (sudden cardiac death), and I46.9 (cardiac arrest, unspecified). These diagnoses were specifically assigned to children who underwent chest compressions and assisted ventilation during their hospital stay. This study included the patients who also had codes from the International Statistical Classification of Diseases and Related Health Problems, 9th Revision, Clinical Modification (ICD-9-CM): 99.60 (cardiopulmonary resuscitation, not elsewhere classified), 99.63 (closed chest cardiac massage), and 99.62 (other electrical countershock of the heart). The data of patients who did not meet the predetermined criteria for assigning a disease code were excluded from the study.

### Data collections

The demographic information regarding the patient upon hospitalization includes their age, gender, province of hospitalization, and the size of the hospital. Detailed hospitalization data encompasses the principal diagnosis, along with the precise date, month, and year of admission. The study utilizes the associated diagnosis of cardiac arrest, categorized according to the Clinical Practice Guidelines for Pediatric Advanced Life Support [17] and extracted from database according to the ICD-10-TM coding system as follows: 1) hypovolemia (ICD-10-TM: E86, T79.4, and T81.1); 2) hypoxia (ICD-10-TM: R09.0 and R09.2); 3) metabolic acidosis (ICD-10-TM: E87.2); 4) Hypoglycemia (ICD-10-TM: E16.1 and E16.2); 5) Hypokalemia/hyperkalemia (ICD-10-TM: E87.6 and E87.5); 6) Hypothermia (ICD-10-TM: T68); 7) Tension pneumothorax (ICD-10-TM: J93.0 to J93.9); 8) Pulmonary embolism (ICD-10-TM: I26.0 to I26.9).

### Outcome measurement

The primary complications involved multi-organ dysfunction, as documented in the complications section of the medical record. Because the NHSO registry does not include the timing of these diagnoses, they represent organ dysfunction identified during the hospitalization and may have occurred before, during, or after the cardiac arrest event. Diagnoses of multi-organ dysfunction were identified using the ICD-10-TM comorbidity codes which included: 1) acute respiratory failure (ICD-10-TM codes: J80 and J96); 2) acute renal failure (ICD-10-TM codes: N17); 3) acute liver failure (ICD-10-TM codes: K72); 4) disseminated intravascular coagulation (DIC), indicative of hematologic involvement (ICD-10-TM code: D65); and 5) anoxic brain damage (ICD-10-TM codes: G93.1, G93.4, and R40).

The length of a hospital stay is calculated in days by subtracting the admission date from the discharge date, regardless of the status at discharge. Discharge status was documented at the time of discharge and categorized into two groups: deceased and survivors. Children who survived their hospital discharge were classified as having achieved a successful survival outcome.

Long-term survival outcomes were obtained from the Civil Registration Section of the Department of Provincial Administration, Ministry of Interior. In accordance with Thai law, all deaths must be reported to the Ministry of the Interior within a 24-hour timeframe. This reporting process has been optimized through electronic linkage to the NHSO database using the individual’s National Identity Card number. Data on survival status was collected through the end of December 2023. For patients experiencing multiple cardiac arrest incidents, only the most recent occurrence was documented and analyzed.

### Statistical analyses

Continuous variable data were reported as means accompanied by standard deviations (SD), while categorical data were presented as both numbers and percentages. The Chi-square test was used to thoroughly evaluate the relationships among categorical variables. A t-test was used to compare the means of two independent continuous variables. Furthermore, the incidence of admissions was calculated based on children’s hospitalizations and expressed as rates per 1,000 individuals in the population. Cochran-Armitage test for trend was used to investigate trends of incidence and mortality across the study period.

In survival time analysis, the observation period represented the date of discharge from hospitals to the endpoint (date of death or December 2023). At the endpoint, patients were considered to have survived if no data indicating death was reported. Survival analysis was conducted using the Kaplan-Meier method, with comparisons between age groups made via the log-rank test. The mean survival time was calculated using the restricted mean survival time method. Additionally, the Cox proportional hazards model was used to assess factors associated with long-term mortality following hospital discharge. A simple proportional hazards Cox regression analysis was performed to calculate crude hazard ratios (HRs), and a multiple Cox proportional hazards regression analysis was performed to evaluate the adjusted hazard ratios (aHRs) in conjunction with 95% confidence intervals (CIs). Covariates included in the Cox proportional models were demographic data, associated diagnosis of cardiac arrest, and organ dysfunction. Variables with p-values less than 0.2 from univariate Cox regression analysis were included in a multivariate Cox regression model. Statistical significance was indicated by a p-value of < 0.05. All statistical analyses were conducted using Stata version 18 (Stata Corp LLC, College Station, TX, US).

## Results

### Incidence of IHCA

**Fig 1:**
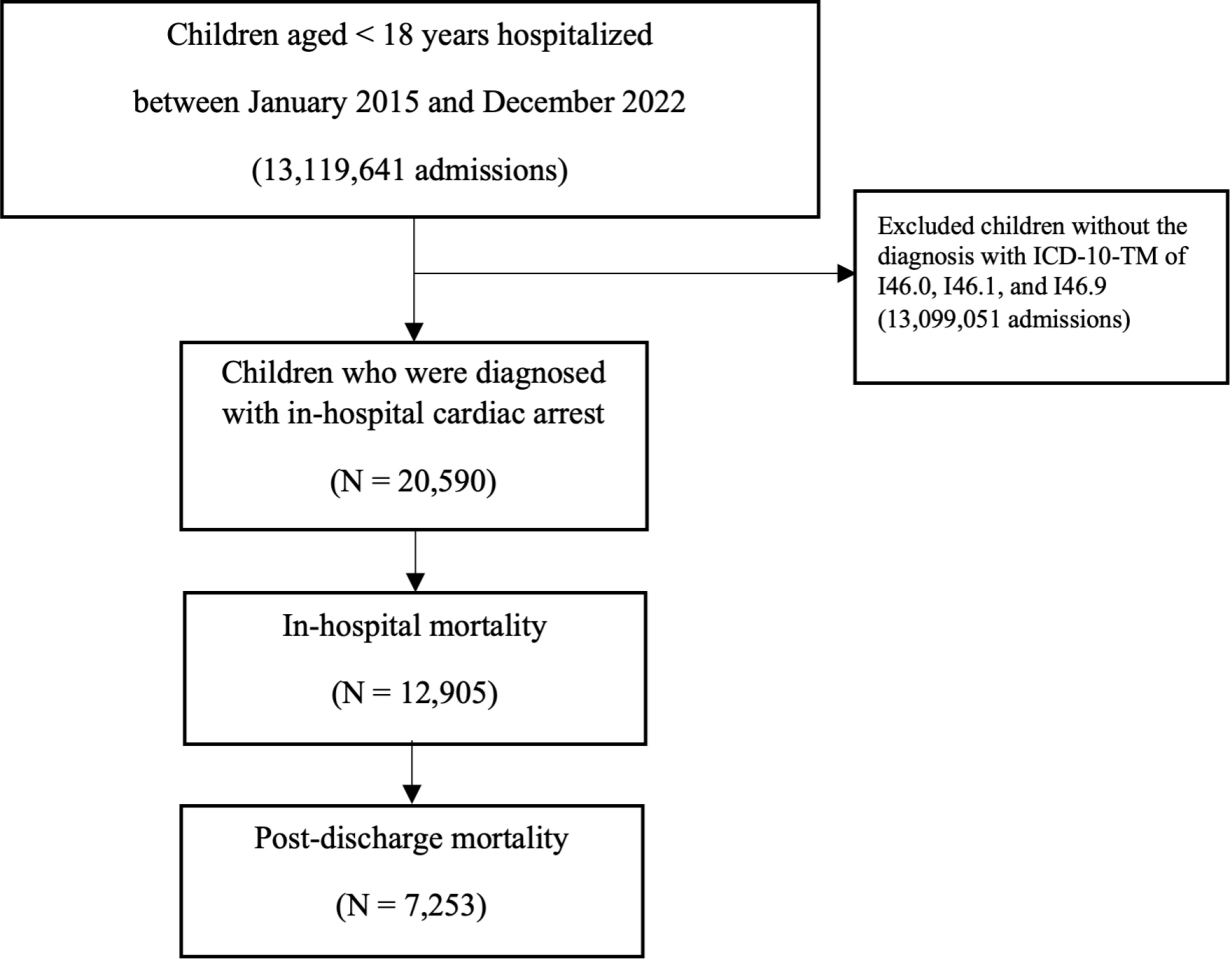
Flow diagram summarizes total pediatric hospitalized with in-hospital cardiac arrests, in-hospital mortality, and post-discharge mortality between January 2015 and December 2022.

Between January 2015 and December 2022, there were between 1,321,335 and 1,845,969 pediatric admissions per year in Thailand, totaling 13,119,641 admissions. A flow diagram illustrating the number of pediatric admissions assessed, the identification of IHCA cases, exclusions based on coding criteria, and the final cohorts included in each analysis has been added as Fig 1. Across this eight‐year span, a total of 20,590 IHCA admissions were identified, corresponding to an overall incidence of 1.57 per 1,000 admissions. Year‐by‐year analysis showed that the incidence was highest in 2015 and 2016 (1.8 per 1,000 admissions), then gradually declined to 1.2 per 1,000 in 2022 (p < 0.001). Over the entire study, the number of IHCA‐related mortality per 1,000 admissions decreased from 1.2 in 2015 to 0.8 in 2022 (p < 0.001) (Table 1).

**Table 1:** Incidence and mortality rate of in-hospital cardiac arrest among Thai children hospitalized during 2015-2022.

Year	Number of All Admissions	Number of IHCA Admissions	Incidence of IHCA (/1,000 admission)	Number of IHCA patients Deaths	Mortality rate of IHCA patients (/1,000 admission)
2015	1,792,423	3,255	1.8	2,071	1.2
2016	1,839,387	3,262	1.8	2,050	1.1
2017	1,728,518	2,882	1.7	1,802	1.0
2018	1,845,969	2,850	1.5	1,778	1.0
2019	1,807,718	2,627	1.5	1,676	0.9
2020	1,425,323	2,136	1.5	1,323	0.9
2021	1,321,335	1,913	1.4	1,171	0.9
2022	1,358,968	1,665	1.2	1,034	0.8

IHCA; in-hospital cardiac arrest.

### Patient characteristics

Demographic and clinical characteristics of the 20,590 IHCA admissions are shown in Table 2. Most patients were male (58.4%, n = 12,022). Neonatal younger than 1 month accounted for nearly half of all IHCA cases (40.9%, n = 8,429), and they had a comparatively higher survival rate (48.9%) compared to older children (p < 0.001). Regional distribution was also significantly different between survivors and non-survivors (p < 0.001), with the Northeast region of Thailand having the largest proportion of IHCA events (30.4%, n = 6,255). Over two-thirds of IHCA cases (66.8%, n = 13,761) occurred in tertiary-level hospitals, and this distribution had a significant association with outcome (p < 0.001) (Table 2).

**Table 2:** Characteristics of Thai children experiencing in-hospital cardiac arrest, categorized by discharge status.

Characteristics	Total (n = 20,590)	Survivors (n = 7,685)	Non-survivors (n = 12,905)	p-value
**Sex, n (%)**				0.075
Male	12,022 (58.4)	4,548 (59.2)	7,474 (57.9)	
Female	8,568 (41.6)	3,137 (40.8)	5,431 (42.1)	
**Age group, n (%)**				<0.001
0- < 1 month	8,429 (40.9)	3,756 (48.9)	4,673 (36.2)	
1 month- < 1 year	2,949 (14.3)	1,040 (13.5)	1,909 (14.8)	
1- < 5 years	2,827 (13.7)	971 (12.6)	1,856 (14.4)	
5- < 12 years	2,375 (11.5)	739 (9.6)	1,636 (12.7)	
12- < 18 years	4,010 (19.5)	1,179 (15.3)	2,831 (21.9)	
**Region, n (%)**				<0.001
Bangkok	2,542 (12.4)	951 (12.4)	1,591 (12.3)	
Central	4,814 (23.4)	1,649 (21.5)	3,165 (24.5)	
Northeast	6,255 (30.4)	2,267 (29.5)	3,988 (30.9)	
North	2,941 (14.3)	1,234 (16.1)	1,707 (13.2)	
South	4,038 (19.6)	1,584 (20.6)	2,454 (19.0)	
**Hospital level, n (%)**				<0.001
Primary	515 (2.5)	295 (3.8)	220 (1.7)	
Secondary level	5,933 (28.8)	2,577 (33.5)	3,356 (26.0)	
Tertiary	13,761 (66.8)	4,690 (61.0)	9,071 (70.3)	
Private	381 (1.9)	123 (1.6)	258 (2.0)	
**Associated diagnoses, n (%)**				
Hypovolemia	584 (2.8)	97 (1.3)	487 (3.8)	<0.001
Hypoxia	316 (1.5)	99 (1.3)	217 (1.7)	0.026
Metabolic acidosis	2,460 (12.0)	542 (7.1)	1,918 (14.9)	<0.001
Hypoglycemia	1,001 (4.9)	169 (2.2)	832 (6.5)	<0.001
Hypokalemia/hyperkalemia	5,261 (25.6)	1,697 (22.1)	3,564 (27.6)	<0.001
Hypothermia	40 (0.2)	06 (0.1)	34 (0.3)	0.003
Tension pneumothorax	704 (3.4)	194 (2.5)	510 (4.0)	<0.001
Pulmonary embolism	34 (0.2)	9 (0.1)	25 (0.2)	0.190
**Organ dysfunction, n (%)**				
Acute respiratory failure	6,250 (30.4)	1,688 (22.0)	4,562 (35.4)	<0.001
Acute renal failure	2,299 (11.2)	474 (6.2)	1,825 (14.1)	<0.001
Acute liver failure	664 (3.2)	120 (1.6)	544 (4.2)	<0.001
Disseminated Intravascular coagulation	1,263 (6.1)	195 (2.5)	1,068 (8.3)	<0.001
Anoxic brain damage	965 (4.7)	408 (5.3)	557 (4.3)	0.001
**Length of stay (days),** mean ± SD	17.6 ± 35.5	27.3 ± 41.4	11.9 ± 29.9	<0.001

### In-hospital mortality

Overall, in‐hospital mortality among the 20,590 IHCA admissions was 62.7% (n = 12,905). Several associated diagnoses with cardiac arrest were notably more frequent among non‐survivors, including metabolic acidosis (14.9% vs. 7.1% among survivors; p < 0.001), hypoglycemia (17.0% vs. 10.8%; p < 0.001), and electrolyte imbalances (hypo/hyperkalemia at 27.6% vs. 22.1%; p < 0.001). Organ dysfunctions such as acute respiratory failure, acute renal failure, acute liver failure, and DIC were significantly prevalent among the non-survivor groups. This highlights the critical impact of these conditions on patient outcomes. The length of stay (LOS) differed markedly: survivors had a mean LOS of 27.3 ± 41.4 days, whereas non‐survivors had only 11.9 ± 29.9 days (p < 0.001) (Table 2).

### Long-term survival after hospital discharge

**Fig 2:**
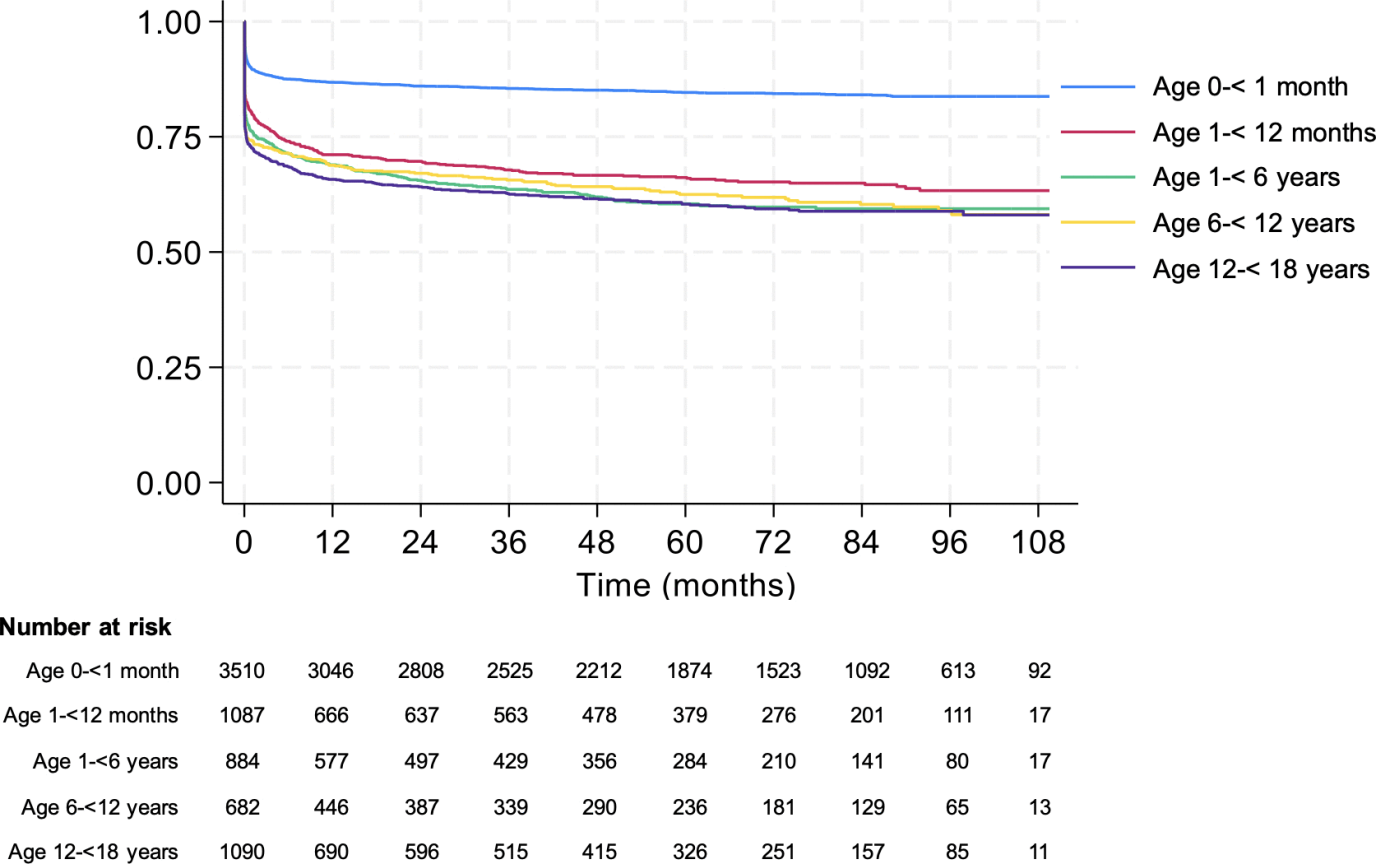
Cumulative survival rates in months for patients after in-hospital cardiac arrest using Kaplan-Meier curve.

Long-term survival data following discharge were obtained from 7,253 cases, indicating that 2,149 (29.6%) children subsequently died post-discharge. Kaplan-Meier curves illustrated that neonates (0 to under 1 month of age) had the most favorable post-discharge survival probabilities, while older pediatric populations, particularly those aged 12 to under 18 years, exhibited lower survival trajectories (Fig 2). At the end of the follow-up period, there were 2,149 deaths among 7,253 survivors at hospital discharge (29.6%). Neonates had mean survival time of 94.6 months (95% CI 93.3–95.9), which was significantly longer than children aged 1- < 12 months (62.4 months; 95%CI 59.3–65.6), 1- < 5 years (65.8 months; 95% CI 62.3–69.3), 5- < 12 years (68.1 months; 95% CI 64.2–72.0), or 12- < 18 years (65.6 months; 95% CI 62.4–68.7). Children with metabolic acidosis and hypoglycemia had substantially lower mean survival times post‐discharge than those without these conditions, mean survival time 45.8 (95% CI 41.4–50.4; p < 0.001) and 42.2 months (95% CI 34.4–50.1; p < 0.001), respectively (Table 3).

**Table 3:** Mean Survival Time Following Hospital Discharge for In-Hospital Cardiac Arrest in Thai Children.

Factors	Total (n = 7,253)	Non-survivors (n = 2,149)	Mean survival time	95%CI	P-value
**Age group**					<0.001
0- < 1 month	3,510 (48.4)	543 (15.5)	94.6	93.3, 95.9	
1- < 12 months	1,087 (15.0)	495 (45.5)	62.4	59.3, 65.6	
1- < 6 years	884 (12.2)	373 (42.2)	65.8	62.3, 69.3	
6- < 12 years	682 (9.4)	281 (41.2)	66.4	62.5, 70.4	
12- < 18 years	1,090 (15.0)	457 (41.9)	65.6	62.4, 68.7	
**Hospital level**					
Primary	263 (3.6)	102 (38.8)	68.0	61.6, 74.3	<0.001
Secondary level	2,399 (33.1)	788 (32.9)	75.5	73.5, 77.6	
Tertiary	4,479 (61.8)	1,222 (27.3)	82.2	80.8, 83.6	
Private	112 (1.5)	37 (33.0)	74.6	65.3, 83.9	
**Associated diagnosis**					
Hypovolemia					
Yes	93 (1.3)	44 (47.3)	59.0	48.5, 69.6	<0.001
No	7,160 (98.7)	2,105 (29.4)	79.7	78.5, 80.8	
Hypoxia					
Yes	91 (1.3)	31 (34.1)	70.8	61.0, 80.5	0.383
No	7,162 (98.7)	2,118 (29.6)	79.5	78.4, 80.6	
Metabolic acidosis					
Yes	502 (6.9)	302 (60.2)	45.8	41.2, 50.4	<0.001
No	6,751 (93.1)	1,847 (27.4)	81.9	80.8, 83.1	
Hypoglycemia					
Yes	162 (2.2)	103 (63.6)	42.2	34.4, 50.1	<0.001
No	7,091 (97.8)	2,046 (28.9)	80.3	79.1, 81.4	
Hypokalemia/hyperkalemia					
Yes	1,593 (22.0)	651 (40.9)	67.3	64.7, 69.9	<0.001
No	5,660 (78.0)	1,498 (26.5)	82.7	81.5, 84.0	
Hypothermia					
Yes	06 (0.1)	3 (50.0)	30.9	6.3, 55.4	0.174
No	7,247 (99.9)	2,146 (29.6)	79.5	78.3, 80.6	
Tension pneumothorax					
Yes	181 (2.5)	61 (33.7)	74.3	66.9, 81.7	0.167
No	7,072 (97.5)	2,088 (29.5)	79.6	78.4, 80.7	
Pulmonary embolism					
Yes	8 (0.1)	2 (25.0)	82.3	49.4, 115.2	0.863
No	7,245 (99.9)	2,147 (29.6)	79.4	78.3, 80.6	
**Organ dysfunction**					
Acute respiratory failure					
Yes	1,577 (21.7)	777 (49.3)	58.1	55.4, 60.7	<0.001
No	5,676 (78.3)	1,372 (24.2)	85.4	84.2, 86.5	
Acute renal failure					
Yes	442 (6.1)	229 (51.8)	54.8	49.8, 59.9	<0.001
No	6,811 (93.9)	1,920 (28.2)	81.0	79.8, 82.1	
Acute liver failure	114 (1.6)	68 (59.7)	45.3	35.8, 54.8	<0.001
Yes					
No	7,139 (98.4)	2,081 (29.2)	80.0	78.8, 81.1	
Disseminated Intravascular coagulation					
Yes	191 (2.6)	114 (59.7)	44.7	37.4, 52.0	<0.001
No	7,062 (97.4)	2,035 (28.8)	80.3	79.2, 81.5	
Anoxic brain damage					
Yes	360 (5.0)	184 (51.1)	56.3	50.8, 61.8	<0.001
No	6,893 (95.0)	1,965 (28.5)	80.6	79.5, 81.8	

Multivariable analyses identified age ≥ 1 month as a significant independent predictor of post-discharge mortality. In comparison to neonates, children aged 1 to <5 years exhibited aHR of 3.07 (95% CI 2.65–3.56; p < 0.001), while those aged 5 to <12 years demonstrated an aHR of 3.17 (95% CI 2.71–3.71; p < 0.001), and adolescents aged 12 to <18 years showed an aHR of 3.37 (95% CI 2.94–3.87; p < 0.001). Among the various associated diagnoses of cardiac arrest, hypoglycemia was identified as a strong predictor of mortality, with an aHR of 1.54 (95% CI 1.25–1.89; p < 0.001), followed closely by metabolic acidosis, which had an aHR of 1.50 (95% CI 1.32–1.71; p < 0.001). Regarding organ dysfunction, DIC was associated with an aHR of 1.51 (95% CI 1.24–1.85; p < 0.001), whereas acute liver failure associated with an aHR of 1.42 (1.10–1.84; p = 0.008). Importantly, anoxic brain damage also demonstrated a significant association with an aHR of 1.23 (95% CI 1.05–1.44; p = 0.009) (Table 4).

**Table 4:** Factors associated with mortality after discharge from in-hospital cardiac arrest (N = 7,253 cases).

Characteristics	Crude HR	Adjusted HR	95%CI	p-value
**Age group**				<0.001
0- < 1 month	Reference			
1- < 12 months	3.45	2.98	2.60, 3.42	
1- < 5 years	3.19	3.07	2.65, 3.56	
5- < 12 years	3.14	3.17	2.71, 3.71	
12- < 18 years	3.25	3.37	2.94, 3.87	
**Associated diagnosis**				
Hypovolemia	1.81	1.19	0.88, 1.60	0.265
Metabolic acidosis	2.84	1.50	1.32, 1.71	<0.001
Hypoglycemia	2.85	1.54	1.25, 1.89	<0.001
Hypokalemia/hyperkalemia	1.66	0.93	0.83, 1.03	0.149
Hypothermia	2.11	0.81	0.26, 2.53	0.717
Tension pneumothorax	1.19	0.77	0.60, 1.00	0.053
**Organ dysfunction**				
Acute respiratory failure	2.42	1.13	1.02, 1.25	0.024
Acute renal failure	2.20	1.22	1.05, 1.43	0.012
Acute liver failure	2.62	1.42	1.10, 1.84	0.008
Disseminated Intravascular coagulation	2.63	1.51	1.24, 1.85	<0.001
Anoxic brain damage	2.00	1.23	1.05, 1.44	0.009

HR; hazard ratio.

## Discussion

Our comprehensive eight-year study of a large population found that the overall incidence of IHCA was 1.57 per 1,000 admissions, with an in-hospital mortality rate of 62.7%. These findings highlight the significant clinical burden and the urgent need for continued efforts to reduce mortality in hospitalized children. In our nationwide dataset, both the incidence and mortality of pediatric IHCA declined over time. Although our database did not capture the drivers of this trend, possible contributors include improved pediatric critical care capacity, earlier recognition of at-risk patients, and broader resuscitation training. Future studies linking administrative data with process-of-care measures will be needed to confirm these explanations. In our cohort, survival to hospital discharge was 37.3%, which is notably similar to previously reported from multi‐center registries in high‐income countries, but our observed mortality rates are higher than those in some resource‐rich settings, where survival to hospital discharge has been reported to be 39.8% [18]. This similarity suggests that, despite differences in healthcare structure and resource distribution, short-term survival outcomes following pediatric IHCA in Thailand are broadly comparable to those reported internationally.

Clear age-related variation in post-discharge mortality was observed in this study. Neonates younger than one month had the lowest long-term mortality and were therefore used as the reference group. In contrast, infants between 1 and 12 months of age showed a substantially higher risk of death after discharge, with adjusted hazard ratios closely approximating those seen in older pediatric age groups. This pattern indicates that the apparent survival advantage is primarily limited to the neonatal period rather than encompassing infancy as a whole. Favorable neonatal outcomes may be attributable to the highly structured nature of neonatal intensive care, including continuous physiologic surveillance and standardized approaches to metabolic and hemodynamic management [19,20]. Conversely, the higher mortality among infants and older children may reflect greater heterogeneity in arrest causes, increasing clinical complexity, and a higher burden of underlying conditions, all of which have been linked to poorer outcomes following pediatric cardiac arrest [21].

A striking finding from our results is the elevated risk associated with metabolic abnormalities, particularly metabolic acidosis and hypoglycemia. Both conditions are strong indicators of suboptimal organ function and can represent underlying metabolic or endocrine issues [22]. These results aligned with prior work show that metabolic derangements can precipitate or prolong circulatory collapse [23]. Early identification and aggressive correction of these abnormalities could reduce IHCA incidence and improve survival [1].

Further underpinning this conclusion, DIC increased long‐term mortality risk. This is consistent with literature that pinpoints DIC as a reflection of severe systemic stress, such as sepsis, trauma, or advanced organ failure [24,25]. Our findings also parallel studies demonstrating strong associations between multi‐organ dysfunction and negative outcomes in pediatric critical care [26–28]. Early recognition of these complications in clinical practice remains crucial; however, in our registry-based analysis we could not determine whether they occurred before, during, or after the cardiac arrest event, and they should therefore be interpreted as markers of severe illness among children who experienced IHCA rather than confirmed post-arrest complications. Our study revealed that several characteristics are significantly associated with survival following in-hospital cardiac arrest. Metabolic acidosis was identified as an important factor associated with increased post-discharge mortality consistent with previous research emphasizing its role in deteriorating prognosis by aggravating cardiac dysfunction and lowering the efficacy of resuscitation attempts [29]. Similarly, hypoglycemia emerged as a significant factor consistent with previous research indicating that low glucose levels severely impede neurological recovery and are associated with poor neurological recovery and increased mortality [30]. In terms of organ dysfunction, acute liver failure was significant, supporting previous research showing that severe hepatic involvement is associated with poor outcomes in critically ill children [31,32]. Disseminated intravascular coagulation and anoxic brain damage were also identified as significant negative prognostic factors, consistent with extensive research demonstrating that coagulation disorders and brain injury are strongly associated with poorer outcomes in this population [33,34]. In our dataset, however, these diagnoses should be interpreted as indicators of severe systemic illness among children with IHCA, because the registry does not provide the timing of diagnostic codes. Therefore, we cannot determine whether these organ dysfunctions developed before, during, or after the cardiac arrest, and we are unable to infer a definite causal or temporal sequence despite their strong association with post-discharge mortality.

Comparisons of our findings with international data must be considered within the context of Thailand’s healthcare system, which is relatively well‐developed but still exhibits variability in pediatric critical care capacity among provincial and tertiary hospitals. Several large hospitals have Pediatric Intensive Care Units (PICUs) staffed by trained intensivists, while smaller provincial hospitals may lack such expertise. The higher proportion of IHCA events in tertiary settings (66.8%) underscores that these centers likely receive more severely ill children who are transferred for specialized care.

Advances in early detection, efficient intervention, and high-quality resuscitation have contributed to better survival rates over the decade [8]. Standardizing pediatric early warning scores (PEWS) and rapid-response team activation criteria could expedite the detection of clinical deterioration [35]. In addition, a nationwide push to enhance metabolic monitoring, particularly vigilant glucose and acid‐base assessments, could reduce mortality in at‐risk children [36,37]. Enhanced training in pediatric advanced life support (PALS) for multidisciplinary hospital staff may further improve resuscitation outcomes and limit secondary injuries that contribute to long‐term mortality [38,39].

One of the most striking findings of this study is that nearly 30% of children who survived to hospital discharge died during the follow-up period. Although data are limited, evidence from long-term follow-up studies and systematic overviews suggests that pediatric cardiac arrest survivors often require ongoing care due to dynamic neurologic, cognitive, and functional needs, and that gaps in coordinated follow-up care are common [40]. This outcome highlights the need for robust follow‐up services, including specialized outpatient pediatric cardiology or critical care clinics, alongside community‐level support systems. Many survivors experience new morbidities and functional decline [41]. The need for comprehensive follow-up services is apparent, as research indicates elevated rates of patients being lost to follow-up and gaps in care [42,43]. However, standardized follow-up protocols are lacking, highlighting the need for further research and guideline development.

Although this nationwide analysis benefits from a large, population-based sample, several limitations should be considered. The study relied on administrative data from the National Health Security Office, which limits the depth and granularity of available clinical information and introduces the potential for misclassification or incomplete coding of comorbidities and complications. Because the registry lacks timestamps, we were unable to determine whether certain conditions preceded the cardiac arrest or developed afterward, and some complications may therefore reflect preexisting illness rather than post-arrest organ dysfunction. Detailed resuscitation characteristics including chest compression quality, medication administration, defibrillation, extracorporeal CPR, and post–cardiac arrest care were not available, precluding assessment of resuscitation practices, staff training, or adherence to standardized Utstein reporting elements. Similarly, hospital-level characteristics such as urban or rural location, teaching status, ICU availability, and interdisciplinary resuscitation training could not be evaluated. Information on treatment limitation decisions, including do-not-resuscitate orders, as well as socioeconomic factors and post-discharge care, was also unavailable and may have influenced long-term mortality. Functional and neurological outcomes were not captured, limiting interpretation of survival quality. These constraints underscore the need for future prospective, clinically detailed registries in Thailand that integrate patient comorbidities, hospital resources, resuscitation processes, and long-term neurodevelopmental outcomes to more comprehensively inform quality improvement efforts and survivorship care following pediatric in-hospital cardiac arrest.

## Conclusion

This nationwide study quantified the incidence and mortality of pediatric in-hospital cardiac arrest in Thailand, showing a gradual decline in both over the eight-year period. We described long-term outcomes, with nearly one-third of children who survived to discharge dying during follow-up. During a median follow-up of 67 months, we identified metabolic derangements and organ dysfunction as independent factors associated with post-discharge mortality. These findings directly address our study objectives and provide a foundation for future strategies to improve outcomes in this high-risk population.
